# Antioxidant and Antimicrobial Activity of *Cleome* *droserifolia* (Forssk.) Del. and Its Biological Effects on Redox Status, Immunity, and Gut Microflora

**DOI:** 10.3390/ani11071929

**Published:** 2021-06-28

**Authors:** Nesrein M. Hashem, Mohamed G. Shehata

**Affiliations:** 1 Department of Animal and Fish Production, Faculty of Agriculture, Alexandria University, Alexandria 21545, Egypt; 2 Department of Food Technology, Arid Lands Cultivation Research Institute, City of Scientific Research and Technological Applications (SRTACITY), New Borg El Arab, Alexandria 21545, Egypt; gamalsng@gmail.com

**Keywords:** phytogenic, phenols, antioxidant, antimicrobial activity, immunity

## Abstract

**Simple Summary:**

The antioxidant, antimicrobial, and immunomodulatory activities of the *Cleome droserifolia* (Forssk.) Del. (Cd) shrub were investigated considering the biological activity of its phytogenic compounds. Cd shrub encompasses several phenolic compounds, mainly phenolic acids, such as benzoic acid. The methanolic extract of Cd exhibited strong in vitro antioxidant and antimicrobial activities. Anin vivo study using rabbits as an animal model confirmed the ability of a powder of Cd aerial parts to improve humoral and innate immunity, as well as gastrointestinal microbiota homeostasis. In conclusion, Cd shrub represents a novel source of secondary active metabolites that can be employed as antibiotic alternative in the livestock production field and/or in human pharmaceutical applications.

**Abstract:**

This study aimed to investigate the antioxidant, antimicrobial, and immunomodulatory activities of a *Cleome droserifolia* (Forssk.) Del. (Cd) shoot methanolic extracts considering the biological activity of its phytogenic compounds. For this purpose, the Cd phenolic compounds were detected, and an in vitro evaluation of the antioxidant and antimicrobial activities of the Cd extract was performed. For a biological evaluation, 30 v-line rabbits were randomly distributed into three groups with treatments including: a basal diet without Cd shoots powder supplement (C group) or supplemented with 1.25- (Cdl group) or 2.5 (Cdh group)-mg Cd/kg dry matter (DM). The Cd extract showed a linear scavenging activity for 2,2-diphenyl-1-picrylhydrazyl and 2,2′-azino-bis (3-ethylbenzothiazoline-6-sulfonic acid), with the maximal activity observed at a concentration of 1 mg/mL. A total of 16 phenolic compounds were identified by reverse-phase high-performance liquid chromatography (RP-HPLC) in the Cd methanolic extract, among which benzoic acid, rutin, ellagic acid, *naringenin*, and o-coumaric acid were the major compounds. The methanolic extract of Cd showed inhibitory actions against microbial pathogen species. The in vivo study showed that the two concentrations of Cd significantly improved the redox status of the blood plasma and lysozyme activity. Treatment with Cdh significantly decreased the levels of interleukin-β1 in the blood plasma compared with the control. Moreover, the two concentrations of Cd significantly increased the counts of intestinal and cecal yeast and *Lactobacillus* species and decreased the *Salmonella* and *Coliform* species compared with the control. The aerial parts of the Cd shrub had strong antioxidant, antimicrobial, and immunomodulatory activities, which can improve the overall health status and seem to be related to its impressive range of biologically active phenolic compounds.

## 1. Introduction

Medicinal plants can serve as a natural source of therapeutic drugs, nutraceuticals/food supplements, and feed additives that can be safely used to improve human and animal health. The interest in exploring plants as a new source of different drugs, specifically antimicrobials, has increased in recent decades as an attempt to fight multidrug-resistant bacteria [1,2]. Among the medicinal plants, the *Cleome* genus is one of the largest genera belonging to the family Cleomaceae. This genus encompasses about 180–200 species that are geographically distributed in Egypt, Libya, Palestine, Syria, and other arid and semi-arid regions [3]. Moreover, they are perennial, low, and aromatic cushion-like shrubs with a length of 25–60 cm that exhibit intricately branched stems and broad oval-shaped, three-nerved leaves with swollen glandular hairs [3,4]. The shrubs that belong to this genus have medicinal and ecological importance. *Cleome* genus shrubs are well-known in folk medicine for treating stomachache, skin allergies, and open wounds, as well as for exhibiting anticancer and hepatoprotective properties [5,6,7]. In addition, *Cleome* genus shrubs have shown strong antidiabetic properties; the aqueous extract of *Cleome* has been found to contain a very high percentage of flavonols that showed 63.3% activity, similar to that of the metformin synthetic drug [8]. *Cleome* genus shrubs have antioxidant, antiparasitic, and antimicrobial activities [6]. These biological effects are related to the vast array of secondary metabolites that occur naturally in *Cleome* genus shrubs. Several terpenes, flavonoids, glucosinolates, anthocyanin alkaloids, and polyphenols have been isolated from *Cleome* genus shrubs [3]. Given these biological activities of *Cleome* genus shrubs, additional studies are required to explore the active secondary metabolites of these shrubs and their eligibility to innovate natural feed and food supplements that could be applied for improving animal and human health. Therefore, this study was devoted to the evaluation of the antioxidant, antimicrobial, and immunomodulatory properties of *Cleome droserifolia* (Forssk.) Del., the most famous species among the *Cleome* genus in Egypt, considering phenolic compounds as active secondary metabolites.

## 2. Materials and Methods

### 2.1. In Vitro Evaluation of Cleome droserifolia (Forssk.) Del.

#### 2.1.1. Plant Source and Extraction

Shoots of Cleome droserifolia (Forssk.) Del. (Cd) were collected at “Megerah” Valley, Dahab, the Eastern Desert, South Sina, Egypt during the month of November 2018. The plant was authenticated by the Plant Protection and Biomolecular Diagnosis Department, STR-City, New Borg El-Arab, Egypt. The collected shoots were pooled, dried at 40 °C for 72 h, and milled through a 0.25-mm screen to obtain a fine powder. Representative samples of dried Cd powder were extracted and pooled for evaluating the phenolic content, antioxidant activity, and antimicrobial activity of the plant. Briefly, each 100 g of dried Cd powder was extracted in 1000 mL of hydro-methanolic solution (700-mL methanol and 300-mL water; 70%) at 40 °C for 72 h. The extract was filtered through Whatman No. 1 filter paper (Whatman No. 1, Camlab, Cambridge, UK). The collected filtrate was evaporated at 45 °C to complete dryness, and the residues were then stored at −20 °C for further analyses.

#### 2.1.2. Determination of Total Phenolic and Total Flavonoid Content

The concentrations of total phenols (TPC) and total flavonoids (TFC) of the Cd methanolic extract were colorimetrically (T80 UV/Vis spectrometer PG Instruments LDT, Leicestershire, UK) determined using the Folin–Ciocalteu and aluminum trichloride (AlCl_3_) methods, respectively. Gallic acid (GA) and catechol (CAT) were used as a standard for TPC and TFC measurements, respectively. Results were expressed as mg of GA equivalent/g of the DM extract (mg GA/g DM) and mg of CAT equivalent/g of the DM extract (mg CAT/g DM), as in reference [9].

#### 2.1.3. Determination of Polyphenol Content

All analytical chemicals were gradient grade for the HPLC analysis. All chemicals and standards were purchased from Sigma-Aldrich^®^ (Merck KGaA, Darmstadt, Germany). The polyphenol profile of the Cd extract was assessed using reverse-phase high-performance liquid chromatography (RP-HPLC) in an apparatus coupled with a variable wavelength detector (VWD; Agilent1260 infinity HPLC Series, Agilent, Santa Clara, CA, USA) at a wavelength of 284 nm and fitted with a C18 column (a Kinetex^®^5lJm EVO C18, 106 × 4.6 mm, Phenomenex, Torrance, CA, USA) that was maintained at 35 °C [10,11]. The flow rate of the binary elution phase (A: 0.1% trifluoroacetic acid in water and B: 50% acetonitrile, 49.8% water, and 0.2% trifluoroacetic acid) was kept at 1.0 mL/min using a ternary linear elution gradient (A: 0.2% phosphoric acid, B: 100% methanol, and C: 100% acetonitrile). The measured values were expressed as μg/g of dry weight (μg/g DM) of Cd.

#### 2.1.4. Determination of Antioxidant Activity

The antioxidant activity of the Cd methanolic extract was assessed via a radical scavenging assay using 2,2-diphenyl-1-picrylhydrazyl (DPPH) and 2,2′-azino-bis (3-ethylbenzothiazoline-6-sulfonic acid) (ABTS)-based methods [12]. The antiradical activity of the Cd methanolic extract was determined based on its ability to scavenge the DPPH free radical. In brief, a mixture of 500 μL of the extract at various concentrations with 375 μL of ethanol and 125 μL of DPPH solution (0.02% in ethanol) was prepared. A control containing 875 μL of ethanol and 125 μL of DPPH solution was also prepared. After incubation for 60 min in the dark, the absorbance at 517 nm was measured. The antiradical activity was determined using the following formula: inhibition activity (%) of the DPPH radical = (absorbance (Abs) of the control − Abs of the sample/Abs of the sample) × 100. To determine the scavenging activity of the ABTS radical, two stock solutions were prepared as follows: 7-mM ABTS and 2.4-mM potassium persulfate. The working solution was then prepared by mixing the two stock solutions in equal quantities and allowing them to react for 12 h at room temperature in the dark. The solution was then diluted by mixing 1 mL of the ABTS solution with 60 mL of methanol to obtain an absorbance of 0.802 ± 0.005 units at 734 nm, as assessed using a spectrophotometer (T80 UV/Vis spectrometer PG Instruments LDT, Leicestershire, UK). One milliliter of the Cd methanolic extract was allowed to react with 1 mL of the ABTS solution, and the absorbance was set at 734 nm after 7 min using a spectrophotometer. The antiradical activity was determined using the following formula: inhibition activity of ABTS (%) = (absorbance (Abs) of the control–Abs of the sample/Abs of the control) × 100. The DPPH and ABTS scavenging activities of the Cd extract were compared with the scavenging activity of ascorbic acid.

#### 2.1.5. In Vitro Antimicrobial Activity of *Cleome droserifolia* (Forssk.) Del.

The agar well diffusion method was used to determine the diameters of the inhibition zones of the Cd methanolic extract against five pathogenic strains, including *Staphylococcus aureus* NCTC 10788, *Salmonella senftenberg* ATCC 8400, *Escherichia coli* BA 12296B, *Candida albicans* ATCC MYA-2876, and *Listeria monocytogenes* ATCC 19116. Tests were performed in triplicate, and the results are presented as the mean ± standard error of the mean (SE) [13].

### 2.2. In Vivo Evaluation of Cleome droserifolia (Forssk.) Del.

#### 2.2.1. Experimental Design

Thirty v-line (a maternal line selected for high litter size at weaning) male rabbits (70 days of age) weighing 1224.0 ± 19.91 g at allocation were individually placed in galvanized wire cages (40 × 50 × 35 cm^3^) and housed at the rabbitry of the Laboratory of Rabbit Physiology Research, Agricultural Experimental Station, Faculty of Agriculture, Alexandria University, Alexandria, Egypt. Rabbits were kept under similar management and hygiene conditions. Rabbits were equally allocated into three groups and received the same standard diet supplemented with 0 (C group), 1.25 (Cdl group), or 2.5 (Cdh group) g of Cd shoots powder/kg of DM diet for 4 consecutive weeks (day 0: first day of the treatment and day 30: last day of the treatment). Rabbits were fed on a pellet diet containing (g/kg): 300 alfalfa hay, 230 wheat bran, 180 soybean, 180 barley, 60 yellow maize, 30 molasses, 10 NaCl 10, and CaCo_3_ (18.90% CP and 10.25-MJ/kg digestible energy), covering their daily nutritional requirements according to NRC (1977) [14]. The values of TPC and TFC of the standard diet were 12.81 ± 0.83-mg GA/g DM and 4.16 ± 0.22-mg CAT/g DM, respectively. The half-maximal inhibitory concentration (IC_50_) of the standard diet was 1730 ± 3.12 μg/mL for DPPH and 1492 ± 4.21 μg/mL for ABTS.

Weight, feed consumption, and rectal temperature were recorded weekly for each rabbit. Fecal score was also recorded twice a week for each rabbit and assigned one of the following scores: 1, normal; 2, soft; 3, mixed soft and liquid; and 4, completely liquid [15].

#### 2.2.2. Blood Plasma Hemato-Biochemical Attributes, Redox Status Indicator, and Immunological Variables

Blood samples were collected from the marginal ear vein of rabbit (*n* = 6/group) on experimental days 0 and 30. Each blood sample was divided into two subplots: the first subplot (whole blood) was used to assess the hematological and innate immune variables, and the second subplot was centrifuged at 2000× *g* for 20 min at 4 °C to obtain plasma samples. The plasma samples were stored at −20 °C pending analyses. The counts of red and white blood cells and the packed corpuscular volume were determined. A differential white blood cell count test was also performed to identify the percentage of specific white blood cells [1]. The concentrations of hemoglobin were assessed colorimetrically using commercial kits (Biosystems S.A., Costa Brava, Barcelona, Spain). Phagocytic activity (PA) and the phagocytic index (PI) were determined. A sample of whole blood was mixed (1:1) with *Staphylococcus albus* (1.0 × 10^5^ cells/mL) in phosphate-buffered solution (PBS; pH = 7.2) and incubated for 30 min at 37 °C. A drop of the mixture was transferred to a slide, and a smear was prepared. After drying, the smear was fixed with methanol for 30 min, then processed using Levowitz-Weber staining for 2 min and washed three times with distilled water [16]. Phagocytic cells and engulfed bacteria were counted on a light microscope at a magnification of 100×, and the PA and PI were calculated as follows: PA = percentage of phagocytic cells containing bacterial cells and PI = number of bacterial phagocytosed cells/number of phagocytic cells [16].

The plasma lysozyme activity (LA) was determined using lyophilized *Micrococcus lysodekticus* as the substrate in PBS (pH = 6.4). A plasma sample of 50 µL was mixed with 3 mL of bacterial suspension. The absorbance of the mixture was measured at 570 nm twice at time 0 (directly after plasma addition; A0) and again after incubation of the mixture for 30 min (A30) at 37 °C. The LA was calculated using the following formula: LA = (A0–A30)/A30 [17].

Blood plasma metabolites, including total protein, albumin, and glucose, were determined using commercial kits obtained from Biodiagnostics (Giza, Egypt). The linearity of the methods was up to 10.0 g/dL, 7.0 g/dL, and 500 mg/dL for the total protein content, albumin content, and glucose, respectively. The total antioxidant capacity and malondialdehyde concentration in the plasma were also determined as indicators of the antioxidant and redox status of plasma using commercial kits (Biodiagnostics, Giza, Egypt), according to the instructions of the manufacturer. The linearities of the methods were up to 120 U/mL, 1000 mg/dL, and 2 mM, respectively. The enzyme-linked immunosorbent assay (ELISA) technique was applied to assess the concentrations of immunoglobulin G (IgG), immunoglobulin E (IgE), and immunoglobulin A (IgA) (IBL America Immuno-Biological Laboratories, Inc., Spring Lake Park, MN, USA). According to the manufacturer’s instructions, the sensitivity and specificity of the assays exceeded 96%. Interleukin-1β (IL-1β) was determined in the blood plasma samples (Cat. No. MBS262525, MyBioSource, Inc., San Diego, CA, USA). The lower limit of detection was 5 pg/mL, and the intra- and inter-assay precisions were ≥8% and ≥12%, respectively.

#### 2.2.3. Intestinal and Cecal Microflora Composition

At the end of the experiment (day 30), six rabbits were randomly chosen from each group and were slaughtered [1]. The intestine and cecum were ligated with light twine before separating the cecum from the small intestine. The first part of small intestinal tract and the last part of the cecum were removed and stored in sterile bags at −4 °C. For bacterial enumeration, the intestinal and cecal contents were separately diluted 10-fold (i.e., 10% *w*/*v*) with sterile ice-cold anoxic PBS (0.1 M; pH 7.0) and subsequently homogenized for 3 min in a stomacher. Each homogenate was serially diluted from 10–1 to 10–7. Dilutions were subsequently plated in duplicate on selective agar media for target bacterial groups, and the enumeration results were expressed as colony-forming units (cfu) log 10/g. In particular, Sabouraud Dextrose Agar for yeast counts; de Man, Rogosa and Sharpe (MRS) agar for LAB counts; MacConkey agar media for *coliform* counts; and *Salmonella* and *Shigella* agar plates for *Salmonella* counts were used. Plates were then incubated at 37 °C for 24 to 72 h [18].

**Figure 1:**
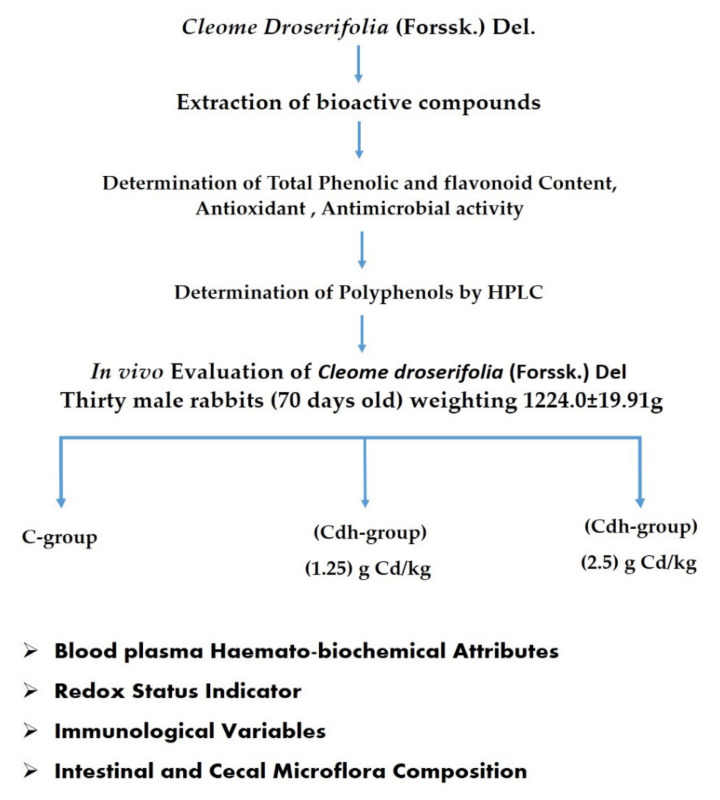
Flow chart of in vitro and in vivo evaluations of *Cleome droserifolia* (Forssk.) Del.

The procedures and methods performed to evaluate Cd’s in vitro and in vivo biological activities are shown in Figure 1.

### 2.3. Statistical Analysis

Statistical Analysis System [19] software was used for analyzing all results. Body weight; feed intake; fecal score; rectal temperature; and hematological, biochemical, and immunological variables were analyzed by the Generalized Linear Model (GLM) method using the following model: yij = μ + Ti + eij, in which yij is the observed value of the dependent variable, μ is the overall mean, Ti is the fixed effect of the *i*th treatment, and eij is the residual error. Comparisons between treatment means were performed using Duncan’s multiple range test. All results were expressed as the mean ± SE. Significance was set at *p* ≤ 0.05.

## 3. Results

### 3.1. RP-HPLC Assessment of Total Phenol and Flavonoid Contents and Phenolic Compound Profile

The values of TPC and TFC of the Cd methanolic extract were 32.55- ± 2.26-mg GA/g DM and 12.78- ±1.86-mg CAT/g DM, respectively (Table 1). The phenolic profile of the Cd methanolic extract detected by RP-HPLC is shown in Table 1. These results revealed that, among the 16 phenolic compounds identified here, the most abundant phenolic compounds, ranging between 1460.62 and 7657.15 μg/g DM, were benzoic acid, rutin, ellagic acid, naringenin, and o-coumaric acid. The second-most abundant phenolic compounds, ranging between 432.14 and 264.06 μg/g DM, were rosmarinic acid, p-hydroxybenzoic acid, resveratrol, kaempferol, quercetin, and ferulic acid. The third-most abundant phenolic compounds were caffeic acid, p-coumaric acid, chlorogenic acid, catechin, syringic acid, and catechin, which were detected in low quantities, ranging between 10.43 and 59.59 μg/g DM.

### 3.2. Antioxidant Activity of the Cd Extract

The antiradical capacity (scavenging activity) of the Cd methanolic extract, as determined by the DPPH and ABTS colorimetric tests, is shown in Table 2. The percent inhibition values of the Cd extract were not much greater than those of the standard antioxidant (ascorbic acid). The Cd extract showed a linear increase in the DPPH and ABTS radical scavenging activities with increasing concentrations, reaching 66.09% ± 1.92% and 81.14% ± 1.26% scavenging activity for DPPH and ABTS, respectively, at concentrations of 1000 μg/mL vs. 87.52% ± 0.62% and 92.44% ± 0.14% for ascorbic acid. The half-maximal inhibitory concentration (IC_50_) of the Cd extract was 470.27 ± 2.24 μg/mL for DPPH and 387.53 ± 3.11 μg/mL for ABTS vs. 16.62 ± 0.91 μg/mL and 14.03 ± 0.67 μg/mL for ascorbic acid, respectively.

### 3.3. In Vitro Antimicrobial Activity

**Figure 2:**
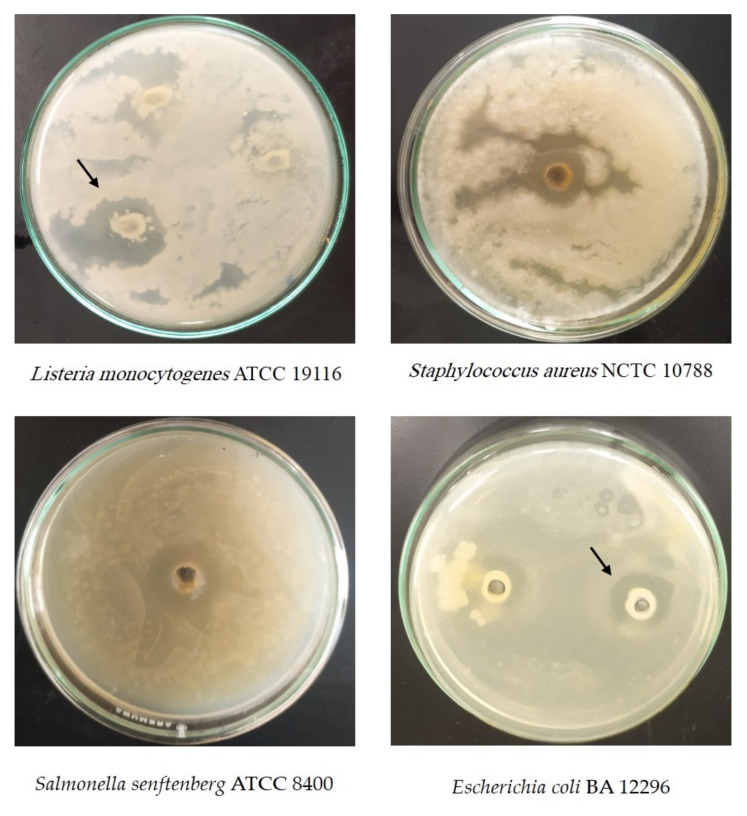
Antimicrobial activity of *Cleome droserifolia* (Forssk.) Del. extract against pathogenic microorganisms.

The methanolic extract of Cd exhibited striking inhibitory actions against *Staphylococcus aureus* NCTC 10788, *Salmonella senftenberg* ATCC 8400, *Escherichia coli* BA 12296, and *Candida albicans* ATCC MAY-2876 (Table 3 and Figure 2). Conversely, the Cd extract was inactive against *Lissteria monocytogenes* ATCC 19116 (Table 3 and Figure 2).

### 3.4. Effect of Treatment on Weight, Feed Intake, and Health Indicators in Rabbits

The treatments with different concentrations of Cd (0, 1.25, or 2.5 g/kg of DM diet) did not affect the overall mean body weight and feed intake of rabbits during the 30-day experimental period (Table 4). The treatment with Cdh tended (*p* < 0.085) to decrease the fecal score compared with the other treatments (Table 4). Compared with the control, the two concentrations of Cd decreased significantly the overall mean rectal temperature (Table 4).

### 3.5. Effect of Treatment on Hemato-Chemistry and Redox Status

The hematological attributes, blood plasma metabolites, and antioxidant activity of rabbits treated with different concentrations of Cd (0, 1.25, or 2.5 g/kg of DM diet) are shown in Table 5. No differences were observed for any of the variables at day 0, confirming the homogeneity of the experimental groups before the beginning of the treatment. At day 30 (the end of the experimental period), the treatment had not affected the hematological attributes or blood plasma metabolites. However, both concentrations of Cd significantly increased the levels of the total antioxidant activity and significantly decreased the levels of malondialdehyde in the blood plasma.

### 3.6. Effect of Treatment on Immune Indicators

#### 3.6.1. Innate Immune System

The innate immune indicators of rabbits treated with different concentrations of Cd (0, 1.25, or 2.5 g/kg of DM diet) are shown in Table 6. No differences were observed for any of the variables at day 0, confirming the homogeneity of the experimental groups before the beginning of the treatment. At day 30 (the end of the experimental period), the treatment had not affected the white blood cell count/differential count, PI, or PA. The treatment with Cdh significantly increased the blood plasma lysozyme activity compared with the C and Cdl treatments. Moreover, the treatment with Cdh significantly decreased the levels of interleukin-β1 in the blood plasma compared with the C treatment, whereas Cdl yielded an intermediate value.

#### 3.6.2. Humoral Immune System

The humoral immune indicators of rabbits treated with different concentrations of Cd (0, 1.25, or 2.5 g/kg of DM diet) are shown in Table 7. No differences were observed for any of the variables at day 0, confirming the homogeneity of the experimental groups before the beginning of the treatment. At day 30 (the end of the experimental period), the two concentrations of Cd had significantly increased the levels of IgG in the blood plasma compared with the control. Conversely, the treatments did not affect the levels of IgA and IgE in the blood plasma.

### 3.7. Intestinal and Cecal Microflora Composition

The gastrointestinal (small intestine and cecum) microflora composition of rabbits treated with different concentrations of Cd (0, 1.25, or 2.5 g/kg of the DM diet) is shown in Table 8. At day 30 (the end of the experimental period), the counts of intestine and cecum *Salmonella* and *Coliform* species were significantly reduced in the Cd-treated groups compared with the control group. The two concentrations of Cd significantly increased the counts of intestinal and cecal yeast and Lactobacillus species compared with the control.

## 4. Discussion

The literature on natural antioxidant utilization as stabilizing and protecting agents for biological macromolecular components is vast. This covers many aspects of their activity against, mainly, reactive oxygen species (ROS), whereas other aspects are less-known or totally ignored [1,12,20]. Despite their remarkable potential for commercial exploitation, species in the *Cleome* genus have attracted interest, and they are currently used as folk medicine for treating stomachaches, cancer, and liver disorders [7,21]. In this study, the values of TPC and TFC obtained for a Cd methanolic extract were close and/or higher than those documented in the literature. For example, the value of TPC obtained in our study was higher than that reported by Aicha et al. [22] in the leaves of Algerian *Cleome L.* varieties (TPC = 35.17-mg GA equivalent/g extract and TFC = 11.35-mg rutin equivalent equivalent/g extract). In another study [21], the TPC was 2.38 mg GA equivalent/g of dried plant extract. It is well-known that the *Cleome* species are an excellent source of phenolic compounds; however, the variations of the TPC and TFC values among the studied samples could be related to many factors, such as the nature of the agro-ecological zones (soil and fertilizers), plant parameters (plant parts and growth phases), extraction method, and assay procedures [23,24].

The antiradical scavenging activity (antioxidant potential) of the Cd methanolic extract was assessed using two colorimetric tests (DPPH and ABTS). Both tests confirmed the strong antioxidant activity of the Cd methanolic extract. These findings are consistent with those of previous studies (DPPH method: reference [25] and ABTS method: reference [26]). In our study, the IC_50_ value of the Cd methanolic extract was less than that of ascorbic acid by about 29-fold. In another study, this difference was only two-fold when the IC_50_ of the extract was compared with that of CAT, which was used as the standard antioxidant [25,27]. In general, these differences in IC_50_ values can be mainly ascribed to variations in the selection of endpoints, the expression of results even within the same method, and the standard antioxidant used. Therefore, comparisons between the values quantified by different laboratories can be quite difficult [28,29].

The strong activity of the Cd extract observed here may be attributed to the presence of phenolic and flavonoid compounds, which are known for their antioxidant activity. Phenolic compounds—in particular, flavonoids and phenolic acids—are able to directly scavenge ROS, such as superoxide anion radicals (O_2−_) and hydroxyl radicals (OH−). They are also able to enhance the expression and activity of antioxidant enzymes via different pathways, such as the nuclear factor erythroid 2-related factor 2 signaling pathway [23,30].

The RP-HPLC analysis performed in our study confirmed the presence of an array of phenolic compounds in the Cd methanolic extract, i.e., phenolic acids, both hydroxybenzoic acid and hydroxycinnamic acid derivatives, and flavonoids. These findings are in line with those obtained by El-Askary et al. (2019) [4], who detected 20 different phenolic compounds in a water extract of Cd, in which phenolic acids (caffeoyl and feruloylquinic acid derivatives) were the major components. Previous studies have reported the presence of numerous active secondary metabolites in *Cleome* species, including phenolic compounds, terpenes, glucosinolates, tannins, and steroids, with different biological activities [3,8,21]. The results of the in vitro antimicrobial activity obtained in our study confirmed the remarkable antimicrobial activity of the Cd methanolic extract against the *Staphylococcus aureus* NCTC 10788, *Salmonella senftenberg* ATCC 8400, *Escherichia coli* BA 12296 *B*, and *Candida albicans* ATCC MYA-2876 pathogen species. The antibacterial activity of the secondary metabolites of the *Cleome* species against both Gram-positive and Gram-negative bacteria has been reported in previous studies [12,31,32].

In this study, the effects of the inclusion of a powder of Cd shoots, to assess its active secondary metabolites, on the health of animals were evaluated using rabbits as a model. Overall, no negative effects of the inclusion of the powder of Cd shoots in the diets of rabbits were observed regarding the hematological parameters, protein and glucose metabolism, and feed intakes. Moreover, all of these variables were in the normal physiological range reported for rabbits. Blood variables can be analyzed to indicate the animal health status and to aid in detecting different nutritional, environmental, or physical stresses [1]. Moreover, linking in vivo results with the antioxidant properties and in vitro antimicrobial activities of the Cd methanolic extract supports the biological activity of the secondary metabolites detected in the Cd methanolic extract. Rabbits that were fed Cd-containing diets had a better redox status and intestinal and cecal microbial homeostasis (lower pathogenic microbes and higher beneficial microbes) than those that were fed the control diet. The antimicrobial activity of the Cd methanolic extract against pathogen species may be related to the presence of many phenolic compounds with antioxidant and antimicrobial activities. Interestingly, the major phenolic compound detected in the Cd methanolic extract was benzoic acid. This phenolic acid and its derivatives can exert antioxidant effects against various types of ROS by reducing their overproduction [33]. These components also possess antibacterial and antifungal proprieties by inhibiting the microbial active uptake of several essential amino acids [34]. Based on such properties, Cd supplements could be used as an adjuvant in treating many oxidative stress-induced diseases without any detected harm. Moreover, the naringenin, rutin, o-coumaric acid, and ellagic acid components detected in the Cd methanolic extract exhibited strong antioxidant and antimicrobial activities [12,31,32,35,36,37,38]. According to the results obtained for the small intestinal and cecal microflora composition, we suggest that the phenolic compounds of Cd can inhibit the growth of pathogenic bacteria (*Salmonella* and *Coliform* species) while stimulating the growth of beneficial microbes (yeast and Lactobacillus species) among the intestinal and cecal microbiota in rabbits, thus optimizing the intestinal microbiota ecosystem. Such enhancements in the intestinal microbiota ecosystem can improve the immune status and digestive health of rabbits.

It is worth noting that the inclusion of a powder of Cd shoots in the diet of rabbits yielded several immunomodulatory effects. These effects occurred mainly through the improvement of the innate immune system, the increase in lysozyme activity, the decrease in the production of the proinflammatory cytokine IL-β1, the improvement of the humoral immune system, and the increase in IgG levels. The immunomodulatory effects of the phenolic compounds of the Cd extract, such as rutin, quercetin, kaempferol, and phenolic acids, have been reported in several studies and depend on many factors, such as the bioavailability and chemical structure of the component [39]. Interestingly, many of the phenolic compounds detected in the Cd methanolic extract are known for their bioavailability because of their increased intestinal absorbance ability. For example, Manach et al. [40] suggested that GA and isoflavones, catechins, flavanones, and quercetin glucosides are among the most well-absorbed phenolic compounds, whereas the least well-absorbed compounds are proanthocyanidins and anthocyanins. In this context, ellagic acid, which was detected in abundance in the Cd methanolic extract, has been found to significantly increase the serum IgM and IgG levels, whereas both IgA and IgE remain unchanged [41]. This phenolic acid also exerted an inhibitory effect on IL-1b secretion in ex vivo and in vivo experiments [41]. In another study, the IgG response was increased after a treatment with a pomegranate extract rich in polyphenols (16.9% GA equivalent/day in calves) [42]. As observed here, Cd-treated rabbits had lower rectal temperatures and gastrointestinal microflora homeostasis, which could be attributed to the enhancement of the immune system function. The levels of the inflammatory factors can be increased as a result of a pathogenic infection and are often associated with elevated body temperatures (rectal temperatures) [43]. In our study, the inclusion of Cd shoots powder in the diets of rabbits decreased the numbers of intestinal and cecal pathogenic bacteria (Salmonella and Coliform). This finding might have explained the decrease in rectal temperature in Cd-treated rabbits. Moreover, the improved lysozyme activity may contribute to the elimination of pathogens because of its enzymatic degradative potential [17]. Finally, increased IgG levels can improve the health of animals in the long term, as these antibodies are responsible for long-term immunological memory [39].

## 5. Conclusions

The results of the present study indicate the impressive range of active phenolic compounds of the shoots of Cd shrub with a multifunctional biological activity. The Cd shrub exhibited strong antioxidant and antimicrobial activities, which were confirmed in vitro and in vivo in our study. These results suggest the possibility of using Cd as an antimicrobial and antioxidant agent. Moreover, this shrub has positive immunomodulatory effects. According to our results, the positive effects of Cd shoots powder on the health status of rabbits can be obtained in vivo at a level of 1.25-mg Cd/kg DM diet. Prospective studies are needed to discover the bioactive natural components of the Cd extract and their specific biological activities.

## Figures and Tables

**Table 1:** Contents of the total phenolic, total flavonoid, and individual phenolic compounds (as detected by reverse-phase high-performance liquid chromatography; RP-HPLC) in the *Cleome droserifolia* (Forssk.) Del. methanolic extract (Cd extract).

Analysis	Content
Total phenols (mean ± SE, mg GA equivalent/g DM)	32.55 ± 0.23
Total flavonoids (mean ± SE, mg CAT equivalent/g DM)	12.78 ± 0.19
Individual detected phenolic compounds (µg/g DM)	
Benzoic acid	7657.15
Rutin	2987.63
Ellagic acid	1641.98
Naringenin	1516.25
o-Coumaric acid	1460.62
Rosmarinicacid	955.27
p-Hydroxybenzoic acid	924.57
Resveratrol	895.77
Kaempferol	778.80
Quercetin	432.14
Ferulic acid	264.06
Caffeic acid	59.59
p-Coumaric acid	39.55
Chlorogenic acid	29.33
Syringic acid	19.29
Catechin	10.43

GA = gallic acid, CAT = catechol, and DM = dry matter.

**Table 2:** Antioxidant activity of the *Cleome droserifolia* (Forssk.) Del. methanolic extract (Cd extract), as assessed by the 2,2-diphenyl-1-picrylhydrazyl (DPPH) and 2,2′-azino-bis (3-ethylbenzothiazoline-6-sulfonic acid (ABTS) tests.

Antioxidant Concentration (µg/mL)	DPPH Scavenging Activity, %		ABTS Scavenging Activity, %	
	Cd Extract	Ascorbic Acid	Cd Extract	Ascorbic Acid
7.81	12.87± 0.91 ^b^	62.66 ± 0.12 ^a^	23.16 ± 0.76 ^b^	64.58 ± 0.24 ^a^
15.6	16.03 ± 0.84 ^b^	75.68 ± 0.45 ^a^	25.09 ± 0.84 ^b^	76.10 ± 0.92 ^a^
31.25	26.88 ± 0.86 ^b^	77.6 ± 0.86 ^a^	34.16 ± 0.92 ^b^	80.21 ± 1.04 ^a^
62.5	31.45 ± 1.12 ^b^	79.11 ± 1.14 ^a^	43.79 ± 1.16 ^b^	82.30 ± 0.86 ^a^
125	34.56 ± 1.24 ^b^	80.20 ± 0.88 ^a^	49.28 ± 1.13 ^b^	85.12 ± 0.45 ^a^
250	46.87 ± 1.16 ^b^	83.2 ± 0.62 ^a^	54.03 ± 0.76 ^b^	88.07 ± 0.93 ^a^
500	53.16 ± 0.85 ^b^	85.4 ± 0.56 ^a^	64.51 ± 0.85 ^b^	89.02 ± 0.88 ^a^
1000	66.09 ± 1.92 ^b^	87.52 ± 0.62 ^a^	81.14 ± 1.26 ^b^	92.44 ± 0.14 ^a^
Half-maximal inhibitory concentration				
(IC_50_) (μg/mL)	470.27 ± 2.24 ^a^	16.62 ± 0.91 ^b^	387.53 ± 3.11 ^a^	14.03 ± 0.67 ^b^

The mean values indicated in the same rows within variable with different superscripts (a and b) were significantly different (*p* < 0.05).

**Table 3:** In vitro antimicrobial activity of *Cleome droserifolia* (Forssk.) Del. against pathogenic microorganisms.

Pathogens Microorganisms	Inhibition Zone (mm)
*Staphylococcus aureus* NCTC 10788	15.63 ± 1.30 ^a^
*Salmonella senftenberg* ATCC 8400	12.70 ± 0.81 ^a^
*Escherichia coli* BA 12296	8.06 ± 1.72 ^b^
*Candida albicans* ATCC MYA-2876	7.16 ± 2.92 ^b^
*Listeria monocytogenes* ATCC 19116	NI

NI, no inhibitory action.

**Table 4:** Body weight, feed intake, fecal score, and rectal temperature of rabbits treated with different concentrations of *Cleome droserifolia* (Forssk.) Del. (Cd) (0: C, Cdl: 1.25 g/kg of DM diet, or Cdh: 2.5 g/kg of DM diet).

Treatment	Variable (Mean± Standard Error of the Mean, *n* = 10/Treatment)			
	Body Weight, g	Feed Intake, g/day	Fecal Score	Rectal Temperature, °C
C	1454 ± 37.01	99.47 ± 17.36	1.19 ± 0.083	39.07 ± 0.112 ^a^
Cdl	1413 ± 33.75	100.06 ± 15.88	1.21 ± 0.073	38.80 ± 0.091 ^b^
Cdh	1393 ± 37.82	99.14 ± 17.31	1.13 ± 0.063	38.74 ± 0.123 ^b^
*p*-Value	0.478	0.968	0.085	0.007

The mean values indicated in the same columns with different superscripts (a and b) were significantly different (*p* < 0.05).

**Table 5:** Hematological attributes, blood plasma metabolites, and antioxidant activity of rabbits treated with different concentrations of *Cleome droserifolia* (Forssk.) Del. (Cd) (0: C, Cdl: 1.25 g/kg of DM diet, or Cdh: 2.5 g/kg of DM diet).

Treatment	Variable (Mean± Standard Error of the Mean, *n* = 6)							
	Red Blood Cell Count (10^6^/mL)	Packed Cell Volume(%)	Hemoglobin, g/dL	Total Protein, g/dL	Albumin, g/dL	Glucose, mg/dL	Total Antioxidant Capacity, Mm/L	Malondialdehyde, nmol/mL
**At day 0**								
C	6.31 ± 1.01	32.67 ± 2.45	10.16 ± 0.32	6.34 ± 0.15	4.37 ± 0.11	93.61 ± 2.27	492.75 ± 0.53	5.23 ± 0.43
Cdl	5.85 ± 1.09	33.05 ± 3.45	10.79 ± 0.58	6.72 ± 0.19	4.01 ± 0.07	91.51 ± 1.15	425.40 ± 1.64	4.92 ± 0.06
Cdh	6.36 ± 0.97	34.45 ± 3.47	10.58 ± 0.79	6.65 ± 0.27	4.08 ± 0.12	93.31 ± 1.24	430.23 ± 1.59	5.20 ± 0.13
*p*-Value	0.764	0.947	0.764	0.742	0.641	0.369	0.4752	0.379
**At day 30**								
C	5.94 ± 1.21	31.35 ± 3.72	10.91 ± 0.67	6.44 ± 0.23	4.37 ± 0.24	91.92 ± 1.01	440.40 ± 0.30 ^b^	4.19 ± 0.25 ^a^
Cdl	5.85 ± 0.98	30.37 ± 1.99	10.91 ± 0.37	6.28 ± 0.24	4.59 ± 0.06	91.56 ± 1.62	444.09 ± 0.95 ^a^	3.83 ± 0.04 ^b^
Cdh	5.61 ± 1.23	30.01± 2.01	9.86± 0.34	6.14 ± 0.11	4.38 ± 0.15	92.41 ± 0.97	443.37 ± 0.92 ^a^	3.73 ± 0.04 ^b^
*p*-Value	0.967	0.641	0.143	0.281	0.287	0.258	0.034	0.002

Mean values indicated in the same columns with different superscripts (a and b) were significantly different (*p* < 0.05).

**Table 6:** Innate immune indicators of rabbits treated with different concentrations of *Cleome droserifolia* (Forssk.) Del. (Cd) (0: C, Cdl: 1.25 g/kg of DM diet, or Cdh: 2.5 g/kg of DM diet).

Treatment	Variable (Mean± Standard Error of the Mean, *n* = 6)								
	White Blood Cells, 10^3^/mm^3^	Lymphocytes, %	Neutrocytes, %	Echinocytes, %	Monocytes, %	Phagocytic Index	Phagocytic Activity, %	Lysozyme Activity, U/mL	Interleukin-β1, pg/mL
**At day 0**									
C	7.29 ± 1.26	39.90 ±1.28	38.85 ± 2.33	12.49 ± 0.78	13.18 ± 2.00	1.94 ± 0.27	24.90 ± 1.24	0.113 ± 0.37	16.91 ± 0.34
Cdl	6.47 ± 0.88	38.88 ± 1.91	33.75 ± 5.29	10.79 ± 1.18	11.65 ± 2.61	2.04 ± 0.13	19.39 ± 0.80	0.092 ± 0.01	15.21 ± 0.72
Cdh	6.33 ± 1.42	38.71 ± 1.99	37.59 ± 3.32	10.12 ± 1.09	12.91 ± 0.69	1.96 ± 0.41	20.95 ± 0.12	0.101 ± 0.01	15.74 ± 0.82
*p*-Value	0.560	0.240	0.338	0.327	0.679	0.804	0.258		0.175
**At day 30**									
C	6.49 ± 0.84	39.56 ± 1.32	32.69 ± 1.35	11.74 ± 0.52	13.22 ± 1.20	2.10 ± 0.35	20.56 ± 1.63	0.104 ± 0.02 ^b^	18.66 ± 0.22 ^a^
Cdl	6.33 ± 0.56	42.01 ± 1.68	33.65 ± 3.05	10.22 ± 0.65	11.28 ± 1.53	2.19 ± 0.54	20.63 ± 1.01	0.106 ± 0.12 ^b^	17.01 ± 0.81 ^ab^
Cdh	6.02 ± 1.40	44.52 ± 1.21	37.63 ± 1.92	11.51 ± 0.89	10.97 ± 1.37	2.49 ± 0.24	21.2 ± 2.01	0.142 ± 0.01 ^a^	15.25 ± 0.92 ^b^
*p*-Value	0.449	0.123	0.236	0.531	0.195	0.446	0.561	0.046	0.001

Mean values indicated in the same columns with different superscripts (a and b) were significantly different (*p* < 0.05).

**Table 7:** Humoral immune indicators (immunoglobulins (Igs)) of rabbits treated with different concentrations of *Cleome droserifolia* (Forssk.) Del. (Cd) (0: C, Cdl: 1.25 g/kg of DM diet, or Cdh: 2.5 g/kg of DM diet).

Treatment	Variable (Mean± Standard Error of the Mean, *n* = 6)		
	IgG, mg/dL	IgA, mg/dL	IgE, mg/dL
**At day 0**	-		
C	981.32 ± 6.65	84.77 ± 2.68	7.73 ± 1.35
Cdl	989.90 ± 10.41	85.79 ± 4.82	6.69 ± 0.67
Cdh	985.74 ± 8.83	88.47 ± 3.45	7.99 ± 0.49
*p*-Value	0.516	0.329	0.1602
**At day 30**	-		
C	974.57 ± 3.84 ^b^	91.78 ± 2.39	7.99 ± 0.78
Cdl	987.91 ± 6.01 ^a^	93.86 ± 2.78	6.88 ± 0.38
Cdh	982.99 ± 7.48 ^a^	93.06 ± 4.26	7.73 ± 0.28
*p*-Value	0.016	0.647	0.359

Mean values indicated in the same columns with different superscripts (a and b) were significantly different (*p* < 0.05). IgG, immunoglobulin G; IgE, immunoglobulin E; and IgA, immunoglobulin A.

**Table 8:** Small intestinal and cecal microflora composition of rabbits treated with different concentrations of *Cleome droserifolia* (Forssk.) Del. (Cd) (0: C, Cdl: 1.25 g/kg of DM diet, or Cdh: 2.5 g/kg of DM diet).

Treatment	Variable (Mean± Standard Error of the Mean, *n* = 6)			
	Yeast	Lactobacillus	*Salmonella*	*Coliform*
Intestinal microflora (log cfu/g)				
C	4.83 ± 0.65 ^b^	6.80 ± 0.91 ^a^	5.96 ± 0.55 ^a^	6.30 ± 0.70 ^a^
Cdl	7.60 ± 0.52 ^a^	8.10 ± 0.94 ^a^	3.10 ± 0.65 ^b^	4.86 ± 0.77 ^a^
Cdh	8.06 ± 0.66 ^a^	8.06 ± 0.70 ^a^	3.13 ± 0.85 ^b^	4.83 ± 0.85 ^a^
Cecal microflora (log cfu/g)				
C	3.56 ± 0.81 ^b^	5.40 ± 0.55 ^a^	7.63 ± 0.86 ^a^	8.13 ± 0.61 ^a^
Cdl	5.60 ± 1.13 ^a^	6.57 ± 1.70 ^a^	5.50 ± 0.45 ^b^	6.06 ± 1.30 ^b^
Cdh	5.27 ± 0.83 ^ab^	6.93 ± 1.53 ^a^	5.34 ± 0.67 ^b^	6.20 ± 0.79 ^b^

Mean values indicated in the same rows with different superscripts (a and b) were significantly different (*p* < 0.05).

## Data Availability

Data of this study are confidential.
